# Maria José Deane por ela mesma e por nós

**DOI:** 10.1590/S0104-59702025000100065

**Published:** 2026-02-09

**Authors:** Laurinda Rosa Maciel, Renata Silva Borges

**Affiliations:** i Tecnologista em saúde pública, Departamento de Arquivo e Documentação/Casa de Oswaldo Cruz/Fiocruz. Rio de Janeiro – RJ – Brasil. laurinda.maciel@fiocruz.br; ii Tecnologista em saúde pública, Departamento de Arquivo e Documentação/Casa de Oswaldo Cruz/Fiocruz. Rio de Janeiro – RJ – Brasil. renata.borges@fiocruz.br

**Keywords:** Maria José von Paumgartten Deane (1916-1995, Ensino, Doenças endêmicas, Família, Feminino, Maria José von Paumgartten Deane (1916-1995, Teaching, Endemic diseases, Family, Female

## Abstract

O texto apresenta e transcreve parte de uma entrevista com Maria José von Paumgartten Deane sobre sua trajetória pessoal e profissional, realizada em 1989, como parte do “Projeto Memória de Manguinhos”, da Casa de Oswaldo Cruz. A depoente relata sua experiência na área científica a partir dos anos 1930, em um contexto predominantemente masculino, e as estratégias de afirmação feminina, narrando ainda as vivências de trabalho com Evandro Chagas, viagens científicas e a parceria de vida com Leônidas de Mello Deane.

## Abstract (en)

The paper presents and transcribes excerpts from an interview with Maria José von Paumgartten Deane in 1989 on her personal and professional trajectory, as part of Casa de Oswaldo Cruz’s “Projeto Memória de Manguinhos.” The interviewee shares her experience in the field of science from the 1930s onward within a predominantly masculine context, as well as strategies for female affirmation, and also describes her experiences working with Evandro Chagas, scientific travels and lifelong partnership with Leônidas de Mello Deane.

É possível conhecer uma versão de Maria José von Paumgartten Deane, ou Maria Deane, uma cientista brasileira, mulher e mãe, pelo que contou sobre si mesma na entrevista realizada por Flávio Taveira e Rose Goldschmidt, em 12 de setembro de 1989,^1^ para o projeto “Memória de Manguinhos”.

Esse não é o único testemunho relacionado à vida de Maria Deane disponível no Departamento de Arquivo e Documentação, da Casa de Oswaldo Cruz (COC). Outros vestígios de sua atuação se encontram preservados na Base Arch, no conteúdo descritivo do arquivo pessoal de Leônidas Deane, seu companheiro, e na Coleção Leônidas e Maria Deane, que reúne documentação iconográfica sobre a trajetória profissional do casal no “desenvolvimento de pesquisas pioneiras sobre leishmaniose visceral no Pará e em excursão científica ao Noroeste do país, na década de 1930, e no Instituto Oswaldo Cruz, após 1980” (Base Arch, s.d.).

É importante destacar que há poucos arquivos de mulheres envolvidas em atividades científicas ou atuantes no contexto da história das ciências e da saúde, embora muitas tenham atuado na saúde pública brasileira, desde as primeiras décadas do século XX (Mulheres..., s.d.). Isso reforça a necessidade de aumentar a visibilidade feminina nos arquivos por meio da aquisição de conjuntos de documentos de mulheres ou de projetos coordenados e desenvolvidos por elas.^2^

A publicação de entrevistas coletadas com mulheres procura contribuir para ampliar a visibilidade sobre a atuação feminina na ciência e na saúde, divulgando tanto o trabalho de constituição de acervo permanente, por meio de projetos de história oral, quanto a captação e coleta de novos arquivos privados e institucionais que as contemplem.

Nesta entrevista, Maria Deane rememora histórias familiares, sua origem, nascimento e infância em Belém do Pará; a educação formal e informal normalizadora e religiosa, associando sua escolha pela medicina às experiências em uma família de professores; o preconceito de gênero sofrido pelas alunas do curso de medicina, desencorajadas pelos professores a seguir na profissão, pelo fato de serem mulheres; o ingresso nas instituições científicas onde atuou, o trabalho com Leônidas Deane e com Evandro Chagas; a atuação em campanhas sanitárias; o recalque masculino em relação às mulheres que tinham salários maiores que os dos homens, e as implicações da maternidade no ritmo da carreira.

Por fim, conta sobre o apoio incondicional do casal à filha, presa política na Argentina durante a ditadura civil militar brasileira e em países da América Latina. Nessas falas estão evidenciadas as dificuldades e superações de uma mulher que sempre lutou pelo desenvolvimento da ciência e pelo direito à educação e à escolha da profissão. Para ela, as mudanças sociais em prol da igualdade de gênero ocorrem com lentidão devido à mentalidade enraizada socialmente em torno de uma dinâmica de inferiorização da mulher.

## Gravação da entrevista e desdobramentos

A COC realizou o projeto de história oral “Memória de Manguinhos” entre 1986 e 1989, cujo objetivo geral era abordar a história do Instituto Oswaldo Cruz até o episódio conhecido como “massacre de Manguinhos”, em 1970. Foram realizadas mais de trezentas horas de entrevistas com trinta pesquisadores considerados fundamentais para a consolidação do Instituto Oswaldo Cruz (IOC) e da própria Fundação Oswaldo Cruz, como a conhecemos atualmente (Britto, 1991).

Assim, entre as entrevistas realizadas no âmbito desse projeto, temos a de Maria Deane e Leônidas Deane, gravadas respectivamente em 1987 e 1989, e ambos não autorizaram seu acesso ao público depois de finalizadas. Não temos conhecimento das razões que levaram a essa decisão, e durante alguns anos a COC fez tentativas infrutíferas de divulgar seu conteúdo. Legalmente, essa autorização somente poderia se dar por um familiar em linha direta. No caso, o neto Camilo Deane Escobar, filho de Luísa Paumgartten Deane, única filha do casal Deane, assinou o termo de consentimento em 2024.

Maria Deane nasceu em 24 de julho de 1916, em Belém do Pará, onde realizou os primeiros estudos e ingressou na Faculdade de Medicina e Cirurgia do Pará, em 1936. Durante o curso, participou da comissão encarregada de estudos sobre leishmaniose visceral, do Serviço de Estudos de Grandes Endemias (Sege), criado por Evandro Chagas em 1937, no âmbito do IOC. Permaneceu no Sege até 1939, quando se transferiu para a campanha contra o *Anopheles gambiae*, atuando no Ceará e no Rio Grande do Norte – a cidade de Natal encontrava-se infestada pelo mosquito transmissor da malária desde o início da década de 1930 (Britto, 1991, p.55).

Em 1942, assumiu o cargo de assistente do Departamento de Parasitologia, do Serviço Especial de Saúde Pública (Sesp), lotada no Instituto Evandro Chagas, em Belém, que era um órgão de pesquisas e laboratório central do Sesp, onde trabalhou com malária e filariose na Amazônia e no Espírito Santo, entre outros estados. O Instituto Evandro Chagas foi criado em 1936 com o nome Instituto de Patologia Experimental do Norte. Após a morte precoce de Evandro Chagas, o instituto recebeu o nome de seu idealizador^3^ (Brasil, 2006).

No Sesp, Maria Deane foi promovida a chefe da seção de parasitologia e desenvolveu pesquisas sobre verminose e leptospirose. Foi chefe do laboratório de entomologia da Campanha de Erradicação da Malária, do Ministério da Saúde, até 1961, quando ingressou no Instituto de Medicina Tropical, da Universidade de São Paulo (USP), e dedicou boa parte de sua vida a estimular a formação de profissionais de saúde.

Em 1969, organizou o Departamento de Microbiologia e Parasitologia da Faculdade de Medicina de Taubaté, e, em 1971, transferiu-se para Minas Gerais, a fim de exercer atividades no Departamento de Zoologia da universidade desse estado. Em 1976, foi convidada pelo governo venezuelano para organizar o Departamento de Parasitologia, da Faculdade de Ciências da Saúde, na Universidade de Carabobo (Britto, 1991, p.55). Maria Deane muito se dedicou à criação e à consolidação de ensino e pesquisa na área da protozoologia em diferentes instituições.

Maria Deane registrou descobertas sobre leishmaniose visceral, malária e doença de Chagas, além de realizar, em 1984, quando estava no Departamento de Protozoologia, do IOC, a descrição do duplo ciclo do agente etiológico da doença de Chagas, *Trypanosoma cruzi*, em gambás, considerado o mais antigo reservatório do parasito. “Os resultados esclarecem a dinâmica de transmissão oral do parasito por este animal e representam contribuição fundamental para a compreensão da epidemiologia da doença em áreas livres de barbeiros domiciliados” (Levy, 22 jul. 2008).

O que a inquietou foi perceber tantos casos de doença de Chagas em regiões do país onde o barbeiro não era comum. Essa foi a questão que a direcionava como cientista, conforme constatamos na palestra proferida por Luis Hildebrando Pereira da Silva, na 11ª Reunião Anual da Sociedade Brasileira de Protozoologia, poucos meses depois do falecimento de Maria Deane, em 13 de agosto de 1995, no Rio de Janeiro, após alguns anos de tratamento contra um câncer ósseo (Maria..., 1996).

Hildebrando a conheceu na década de 1950, quando Samuel Pessoa convidou o casal Deane para integrar o corpo docente do Departamento de Parasitologia da USP, onde era estagiário de medicina, e sabia tratar-se de cientistas respeitados e já conhecidos no campo (Maria..., 1996). Nutriu por Maria Deane amizade e admiração que perduraram pela vida, chegando a visitá-la várias vezes na residência carioca, e foi testemunha da curiosidade que a movia na busca de respostas para a biologia evolutiva dos parasitas (Maria..., 1996).

Segundo ele, os últimos 15 anos de vida foram os mais profícuos de sua trajetória profissional, após a transferência para o IOC em 1980, como pesquisadora titular do Departamento de Protozoologia, que chefiou mais tarde. Em 1986, foi vice-diretora do IOC e, em 1988, uma das responsáveis pela reestruturação de seu curso de pós-graduação (Britto, 1991, p.55). Até 1994, publicou artigos investigativos sobre o tripanossoma em gambás e, como médica, viajou para cidades no interior do Brasil investigando doenças que até hoje afligem o país e são consideradas problemas de saúde pública (Levy, 22 jul. 2008). O texto que apresentamos a seguir é a transcrição editada do depoimento no qual Maria Deane nos apresenta um pouco de sua vida profissional e pessoal.

**Figura 1:**
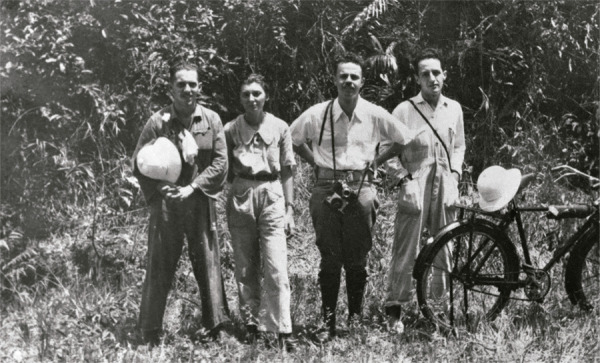
: Leônidas Deane, Maria Deane e Leoberto de Castro Ferreira em trabalho de campo, 1937 (Coleção Leônidas e Maria Deane, Base Arch)

**Figura 2:**
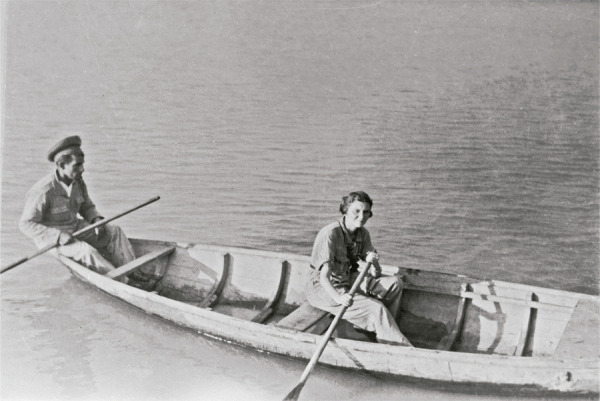
: Maria Deane em barco no rio Paraguai, durante a primeira excursão científica do Instituto Oswaldo Cruz ao Noroeste do Brasil, 1938 (Coleção Leônidas e Maria Deane, Base Arch)

**Figura 3:**
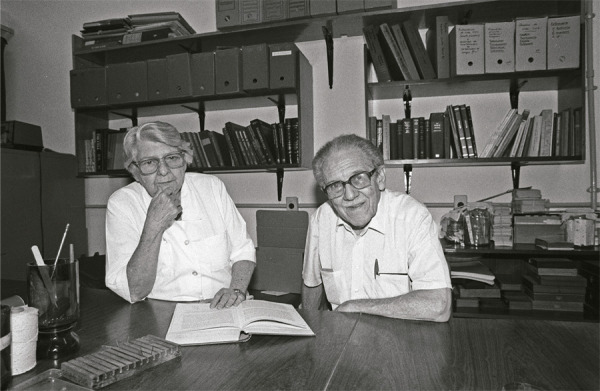
: Leônidas e Maria Deane, em seu laboratório do Departamento de Protozoologia do IOC, Pavilhão Carlos Chagas, em novembro de 1992 (Coleção Leônidas e Maria Deane, Base Arch)

### [Origem familiar e primeiros estudos]

*Nós queríamos que a senhora começasse pelo começo mesmo, que nos contasse onde a senhora nasceu, quando e como foi essa sua primeira infância.*

Foi em Belém do Pará, em 24 de junho de 1916, e meus pais eram brasileiros; mas, do lado do meu pai, o meu avô era austríaco, e minha avó, portuguesa, descendente também de italianos. Quer dizer, uma mistura. Do lado da minha mãe, portugueses. Então, tem aí austríaco, portugueses e italianos... Eu fui a mais velha de cinco; éramos quatro irmãs e um caçula, foi um rapaz, mas uma das minhas irmãs morreu com menos de dois anos. Isso é interessante... lembrar que ela morreu de uma doença que hoje praticamente não mata mais, foi difteria crupe. Naquele tempo era relativamente comum, nós fomos todos isolados e ficamos, então, três meninas e um menino. Meu pai queria um filho macho, não era nada machista, queria um filho homem... Quando meu avô paterno migrou para o Brasil, ele veio naquela onda de migração, porque aquela região no Amazonas, principalmente o Pará, estava num pulso tremendo, no comecinho da riqueza da borracha, e, em parte, ele veio por isso.

*Seu avô veio de onde?*

Da Áustria, e meu avô também tem uma história que eu não gosto muito de contar, mas é história mesmo. Era de uma família com certos recursos e com título de nobreza, era barão, barão von Paumgartten. E eu guardo esse sobrenome à minha revelia, porque quando a gente entra na adolescência começa a enfrentar certas coisas, família e tal, e a repudiar até certas coisas. Então, eu fiz uma tentativa de repudiar esse título, mas não consegui, porém ele veio em parte por causa dessa coisa e, em parte, segundo a história da família, ele veio um pouco fugido da Áustria, porque na família de um certo nível, na Europa toda, os filhos homens eram mais ou menos predestinados para as forças armadas e outro para a Igreja, e meu avô foi destinado ao Exército, tornando-se, oficial. Lá, entrou numa briga[...] Antes, o duelo era permitido, e ele se meteu num duelo, e, como já era proibido, ele teve que fugir, senão ele ia pagar pela briga. Veio para o Brasil e aqui ficou, mas mantendo sempre muitas relações lá com a família. Parte dessas correspondências nós ainda temos.

*O seu avô, antes de falecer, chegou a ter sucesso financeiro no Brasil?*

Não, ele foi professor. Ele era professor de línguas. Eu não o conheci, mas era um homem muito instruído, ficou uma boa parte da biblioteca dele. Ele falava correntemente várias línguas; foi professor de latim e de grego, não teve sucesso financeiro. E a família na Europa também empobreceu. Depois veio a guerra... quando o meu avô morreu, um irmão dele que era embaixador da Áustria nos Estados Unidos ainda quis tomar conta de uma parte dos filhos dele, mas eles não quiseram. E depois meu avô voltou várias vezes à Áustria, para ver a mãe dele. A família tem ainda uma correspondência conservada, mas depois, com a guerra, a gente perdeu completamente os contatos. E, da parte da minha mãe, meu avô era português mesmo, tinha algumas posses, mas também as guerras na Europa empobreceram muitas famílias. Ficou todo mundo pobre [...]

*O Pará, o Norte, é muito diferente, imagino, do Sul. Naquela época, por que tinha esse vínculo tão grande com o exterior?*

Tinha esse vínculo muito grande com a Europa, ia-se mais facilmente à Europa do que ao Rio de Janeiro, e a importação de certos artigos era toda feita diretamente da Europa. Quando eu era menina, a gente usava tecidos vindos da Europa diretamente: caxemira inglesa. Minha mãe, por exemplo, apesar de não sermos ricos, tomava água de elixir importada da França. Agora, aqueles novos ricos daquele tempo, logicamente, não tinham cultura, mas eles tinham meios de mandar os filhos estudar na Europa. Então, se fez lá no Pará, principalmente, uma praça de gente realmente com um certo nível de cultura, educação, devido a esse fato ...

Era relativamente comum você encontrar pessoas que falavam inglês, francês e tal, mas as coisas mudaram muito... depois que o meu avô morreu, as minhas tias organizaram um colégio, guardando uma tradição: meu avô era professor de um colégio estadual, que era de um nível muito bom naquele tempo, porque o Pará teve a sorte, ao mesmo tempo que conseguiu bastante dinheiro, bastante recursos, de ter uns governantes com uma visão muito boa. Então, esses governantes fundaram, por exemplo, o Museu [Emílio] Goeldi; uma escola de química industrial de nível excelente, chamando gente de fora, europeus em geral. Essa parte de educação foi muito cuidada, o colégio estadual era muito bom, de muito bom nível, inclusive, já quando eu fui para o ginasial estadual, o ginásio tinha laboratórios excelentes de física, química, história natural. Então, meu avô foi professor no colégio estadual, e tinha muitos professores estrangeiros, europeus. Naquele tempo, se ensinava latim, grego, alemão. E as minhas tias, depois de algum tempo, organizaram um colégio que se chama Colégio Paumgartten. Foi muito conhecido em Belém, muita gente boa, muitos ex-governadores estudaram no colégio, e eu estudei o primário no colégio das minhas tias... tinha só o primário, tinha o primário completo, ginasial você fazia num ginásio [...]

*E essa parte de religião, como era na família?*

Ah, o pessoal era todo muito católico. Eram católicos. Corre outra história na família – eu não sei até que ponto, fico pensando. Contavam na família, e alguns documentos que a gente tinha mostraram isso: parece que a origem nobre da família do meu avô vem da Guerra dos Trinta Anos, e, segundo parece, a família era de origem judaica. Paumgartten é uma modificação do nome judeu Baumgartten, e eram oito irmãos: seis morreram na luta e dois sobreviveram. Quando houve isso, eles já tinham adotado a religião católica naturalmente para se libertar de fogueiras, o que eu acho que foi uma grande sabedoria. É o cristão novo. Então daí vem a origem, um dos irmãos sobreviventes teve o título de conde e outro de barão. Essa é a história que ficou [risos].

[A Faculdade de Medicina e o início da carreira]

Meu pai teve que interromper os estudos muito cedo. Quando meu avô morreu, ele tinha 16 para 17 anos, precisou deixar os estudos e foi se empregar. Trabalhou como guarda-livros, esse tipo de coisa, uma profissão que hoje não existe mais, hoje ninguém é guarda-livros. E minha mãe, como meu avô materno, vivia indo a Portugal: Brasil-Portugal-Brasil. Meu pai conheceu minha mãe numa dessas viagens que ela fez ao Brasil, e eles demoraram muito a casar, justamente porque o meu pai tinha o encargo da família toda, vários irmãos e a mãe dele etc. Finalmente, casou por procuração, a minha mãe em Portugal e meu pai aqui, durante a Primeira Guerra. Minha mãe tinha se formado na França, tinha feito curso normal, que era o que as mulheres faziam naquele tempo, num colégio em Paris. Ela veio para o Brasil num navio cargueiro no qual ela era a única passageira, sob os cuidados diretos do comandante do navio. Então, ela contava que viviam apavoradas com receio de submarinos alemães etc., etc., mas chegou bem... minha mãe nunca se dedicou ao magistério, minha mãe sempre foi de prendas domésticas.

*[...] Como a senhora se encaminhou para essa carreira tão bem-sucedida?*

Olha, eu não creio que houve uma vocação. Como eu disse a vocês, chegou uma época na minha vida que comecei a pensar mais por mim, me rebelei contra várias coisas, inclusive contra os ensinamentos da Igreja. Hoje eu sou uma pessoa completamente... eu não sou contra religião nenhuma, eu respeito etc., mas eu não tenho, pessoalmente, nenhuma necessidade de qualquer religião, compreende? E isso já vem desde a minha adolescência. Eu me libertei dessas coisas e eu não creio em nenhuma vocação especial. Eu queria, justamente em consequência dessa coisa, ter uma profissão e ganhar a minha vida. Então, eu não tinha muitas opções, eu estava em Belém ainda, e as faculdades em Belém eram medicina, advocacia e engenharia. Eu não dava para engenharia e não queria ser advogada, então fui fazer medicina. Foi por exclusão, digamos, por exclusão, mas evidentemente me encantei com a profissão, com o estudo, e foi isso. Eu lia muito... não fui influenciada pela leitura, não, eu fui influenciada pela minha capacidade de crítica. Quer dizer, comecei a fazer a crítica dos dogmas da Igreja e a não aceitar os dogmas. Até hoje, eu sou uma pessoa que não aceito dogma nenhum. Então, pronto, é só isso, não tem nada, nem influência de leitura, nem ligação com qualquer grupo especialmente político, ideológico contra a religião ou coisa assim, nada disso.

*E dentro de casa, houve essa possibilidade de optar pelo que lhe interessava?*

A minha mãe sempre foi um espírito muito aberto, mas eu lembro que as minhas tias faziam um pouco de objeção. Eu me lembro que, naquele tempo, havia um médico da família, chamava-se doutor Coelho de Souza. Essa figura do médico da família toda, que era enorme, uma porção de filhos e tal. Ele, então, foi consultado pela família se eles deviam deixar que eu entrasse para a escola de medicina. Ele me chamou e disse: “Olha Maria, você quer mesmo fazer?”. Eu digo: “Eu quero”. “Você sabe que você vai ter que examinar cadáveres de homens nus?”[...] me alertou. “E daí?”. Bom, depois da conversa ele disse para família: “Não, deixa ela fazer, não tem problema nenhum”. E assim eu fui fazer o vestibular e entrei. Naquele tempo não havia a competição que há hoje, não sei quantos milhares de candidatos para um número pequeno de vagas; o número era relativamente pequeno, não havia essa disparidade grande. Por coincidência, embora naquela ocasião houvesse muito poucas mulheres médicas no Brasil todo, na minha turma entramos cinco mulheres. Éramos uns sessenta, eu acho[...] por coincidência, cinco Marias [...]

*Havia uma aceitação plena por parte dos homens?*

Entre os colegas, havia. Alguns professores diziam: “Ah, mulher, devia estar em casa, coando café”. Esse tipo de coisa, mas a gente não...

*Pois é, os professores é que faziam pouco caso das alunas?*

Mas eu sempre fui boa estudante, eu gostava.

*A senhora conheceu o doutor Deane ainda estudante. Vocês começaram a namorar...*

... ainda estudantes. Ele era dois anos mais adiantado do que eu. Dois anos mais velho e dois anos mais adiantado.

*Esse encontro com o doutor Deane deve ter marcado muito a sua passagem pela faculdade. Imagino que modificou um pouquinho a sua relação...*

Foi. Ele era muito bom estudante, eu também era uma boa estudante. Tínhamos discussões de grupo, nos conhecemos e um se interessou pelo outro e pronto, normalmente, naturalmente... A gente discutia muito, conversava muito. O Leônidas, por exemplo, sempre gostou muito de estudar essa parte de evolução, o darwinismo etc. e, de certo modo, me introduziu nessa coisa. Ele despertou o meu interesse também para esses aspectos. Eu estava mais voltada, nessa ocasião, para clínica mesmo. Eu tinha um grande encantamento com a clínica e, desde o segundo ano de medicina, eu fui para as enfermarias. Embora o pessoal olhasse um pouco assim, mas eu ia, frequentava, seguia e tal, e assim que me foi permitido – acho que estava no quarto ano – fiz concurso para internato [...] na Santa Casa, que foi um hospital de bastante bom nível, mas já estava decaindo naquela ocasião. Mas tinha sido um hospital de muito bom nível nos tempos áureos da borracha. Belém tinha realmente várias instituições de nível muito bom. A faculdade de medicina já estava decaindo bastante, o hospital também, mas como hospital-escola era bastante bom, e eu tinha muito encantamento pela clínica... tinha clínica e cirurgia, mas eu gostava mais da clínica mesmo. Depois, sob a influência do Leônidas, eu comecei a me interessar também pela parte de trabalho em laboratório. E, depois, quando eu já era formada, quando o Evandro Chagas foi a Belém... Então, por influência do Leônidas, eu entrei para a equipe do Evandro Chagas. Eu ainda era estudante e comecei a viajar para o interior para fazer esse estudo de Calazar. Aí que eu fui sentir um pouco o problema de ser mulher em certas circunstâncias, não na família, que já estava acostumada com o meu comportamento um pouco independente, e também não do grupo de trabalho, que me aceitou muito bem. Mas, na época, achavam que mulher num grupo só de homem... eu nunca liguei para essas coisas, mas sabia. De vez em quando chegavam no meu ouvido comentários assim um pouco [risos]...

*Como a senhora era recebida pelas populações no interior?*

Isso variava... tinha de tudo. Em geral, o pessoal pobre não liga para nenhuma dessas coisas, você vai tratar e não tem problema. Agora, quando você chega assim num grupo com mais dinheiro, mais posição, aí é que começa o preconceito. Isso eu senti um pouco. Realmente, isso nunca me perturbou. Quer dizer, você tem um interesse pelo trabalho, pelo estudo, essas coisas são tão sem importância, não tem importância nenhuma, você não liga mesmo.

### [O trabalho com Evandro Chagas]

*Quando a senhora começou a trabalhar com o doutor Deane nesse grupo do Evandro, era um universo totalmente masculino... porque na universidade tinha colegas.*

Era totalmente masculino. Sempre fui muito bem tratada, muito respeitada pelo grupo, nunca tive qualquer problema no grupo. O Evandro mesmo era um cavalheiro, meus colegas todos cavalheiros, não teve problema nenhum. Nunca dormi com o meu marido antes de me casar, então se querem saber isso, está aí... eu me lembro que uma vez o Evandro estava brincando: “Como é? Quando sai o casamento?”. Eu me lembro muito bem do que eu disse para ele: “Olha, eu não faço questão nenhuma do casamento”. E ele mesmo, o Evandro, disse: “Você está errada Maria, porque nós vivemos numa sociedade onde essas coisas são importantes”. Ele, Evandro Chagas, me disse isso numa roda em que a gente estava conversando e brincando, mas eu nunca me preocupei muito, não [...] Hoje uma moça e um rapaz convivem muito bem como se fossem casados, ninguém repara, está tudo certo, mas naquele tempo havia mesmo. E a gente, mesmo sem pensar no assunto, ter aquele preconceito, também não havia necessidade. Realmente o interesse maior, naquela ocasião, era o trabalho e o estudo, entendeu?

*Perfeitamente. Qual foi o papel do Evandro? Porque o Evandro era uma personalidade e um homem inclusive polêmico. Falam-se coisas muito variadas dele, mas, sem dúvida, parece que era fascinante, não é?*

Era um homem muito inteligente, e dizia-se que era muito mulherengo – e acho que era mesmo –, mas me tratava com o maior cavalheirismo, o maior respeito. Era uma pessoa que..., eu ouso dizer, até com um certo carinho, como se eu fosse filha dele, muito carinho, muito respeito. Ele era jovem, morreu com 35 anos. Não, não tinha idade para ser meu pai, eu tinha vinte e poucos, mas era um homem mais vivido, eu era uma pessoa novinha. Eu tinha, sim, nós todos tínhamos bastante [admiração], era um homem muito generoso, muito inteligente. Depois nós até nos afastamos dele, houve uma cisão no grupo, tivemos uma briga, uma coisa boba e pouco depois ele morreu também. Ele morreu com 35 anos. Nós trabalhamos com ele... Quantos anos? Eu ainda era estudante... [19]39, uns seis anos, por aí, eu me lembro muito bem disso. Ele era um personagem muito interessante, um homem muito bonito também, uma cabeça bonita, ele era muito mais bonito do que o Carlos. Eu conheci muito a mãe do Evandro, dona Íris, quando eu vim para o Rio para trabalhar aqui, trazida pelo Evandro. Eu fiquei morando aqui no Hospital Evandro Chagas, tinha o meu quarto de banho etc. Eu fiz meu internato aí. Nessa ocasião, me dei muito com a então mulher do Evandro, a Agnes Chagas, com a mãe do Evandro, dona Íris, conheci o Carlos Chagas [Filho]. Quando o Evandro veio para Belém, o pai dele tinha morrido fazia tempo. E nos fins de semana era muito frequente, principalmente quando o Evandro estava viajando, a Agnes me levar para o apartamento dele. Almoçava frequentemente na casa da dona Íris, ali na rua Paissandu. Há uns meses, conversando com o Carlinhos Chagas, eu perguntei para ele o que tinha sido feito daquela casa da rua Paissandu. E ele me escreveu um depoimento – eu devo ter por aí – muito interessante sobre a história do pai dele com a mãe dele, a compra da casa da rua Paissandu, tudo isso. Eu tenho escrito por ele. Eu conheci a avó do Evandro, a mãe da dona Íris, e me lembro do orgulho com que dona Íris mostrava as coisas que tinham pertencido ao Carlos Chagas, as medalhas, deve estar tudo aí, não é? [...] Ela me mostrava os documentos, os diplomas, as medalhas, e tinha um livro de visitante. Eu até tenho curiosidade de ver isso porque, naquela ocasião, eu novinha, emocionada, ela me pediu para escrever no livro, e eu me lembro bem o que escrevi: “Espero seguir o seu exemplo”. Quer dizer, o exemplo do pai. Depois eu fiquei pensando: que coisa mais absurda... que pretensão a minha [risos]*.*

*A juventude é assim mesmo [...] essa sua chegada para o Rio de Janeiro, foi a primeira vez que a senhora vinha ao Sul?*

Não, eu tinha vindo criança. Eu tenho duas irmãs nascidas aqui no Rio. Meus pais vieram para o Rio, depois voltaram para o Pará. Minha avó estava muito mal, pediu para o meu pai voltar. Afinal, ele morreu antes dela. Mas a minha mãe nunca perdoou, porque ela adorava o Rio de Janeiro. Eu tenho vaguíssima lembrança [desse tempo]. Eu era muito pequena, eu voltei acho que com uns 5 ou 6 anos. Tenho duas irmãs nascidas aqui.

*Qual foi a impressão que a senhora teve ao chegar na capital federal? Foi alguma coisa importante?*

[...] De certo modo eu conheço, porque eu leio tanto, li tanto sobre grandes cidades, li tanta coisa que é como se eu já conhecesse. Minha mãe tinha um encantamento pelo Rio, então, ela contava muita coisa. Minha mãe era louca pelo Rio. Depois que meu pai morreu, aí já estava trabalhando, ganhando etc., ela veio viver no Rio, morreu aqui no Rio. O sonho dela era viver aqui no Rio. Não tinha deslumbramento, nunca fui muito de me deslumbrar com coisa nenhuma.

*A senhora foi criada em Belém, imagino que aquela experiência de vocês lá naqueles interiores, no mato, naquela selva com o grupo do Evandro, deva ter sido uma coisa bastante forte na sua vida.*

Foi muito interessante, eram aventuras, era uma coisa. Tinha um aspecto meio aventuresco você se meter assim... Realmente, a gente tinha às vezes situações bastante complicadas. Mas as lembranças que eu tenho de todo esse tempo que eu trabalhei no interior com o Evandro e depois de casada com o Leônidas são muito gratas. Foi um tempo realmente muito gostoso[...] de descobertas, não descobertas, mas “descobertinhas”, digamos. De vida, de Brasil, de trabalho, a sensação de que a gente estava fazendo alguma coisa que podia ser útil, se bem que depois a gente fica pensando no que é útil mesmo. Mas, em todo caso, na ocasião, foi muito gostoso, as lembranças que eu tenho são muito gostosas, muito boas, apesar de alguns riscos e muito desconforto. Depois trabalhamos, Leônidas e eu, não só na Amazônia, mas em grande parte do Brasil, no Nordeste todo...

### [A vinda para o Instituto Oswaldo Cruz]

*Quando a senhora veio para o Instituto Oswaldo Cruz, ele tinha alguma importância? Aquele mito do instituto existia para a senhora?*

Tinha, existia muito para o Leônidas e para mim. Eu conheci aqui pessoas que tinham uma importância enorme para nós por meio de estudos e leitura, como, por exemplo, o Henrique Beaurepaire de Aragão, o Julio Muniz, o Arthur Neiva, Costa Lima, essa gente toda que trabalhava aqui. Ainda cheguei a conhecer o velho Adolpho Lutz[...] A Bertha eu não conheci, ela não trabalhava aqui, mas conheci o velho Lutz.

*Quando a senhora veio para o instituto, não tinha nenhuma mulher de peso que a senhora possa mencionar?*

Não, não tinha. Eu trabalhei com o Evandro e com o Walter Oswaldo Cruz e comecei a fazer o Curso de Aplicação em Manguinhos. Mas aí começou a epidemia de malária no Nordeste, o Evandro se engajou e quis que nós fôssemos para lá. Eu fui para lá também, e deixei o curso [...] Aí eu conheci todo esse pessoal que a gente conhecia de trabalho duro e tal.

*Mais uma vez um universo totalmente masculino aqui dentro [...] e a senhora tinha uma relação com as esposas deles, com as famílias?*

Um pouco, nem tanto assim. Tinha com a Agnes Chagas, a mulher do Evandro; eu conheci, mas muito de passagem, a mulher do Carlos Chagas, mas não tinha nenhuma intimidade, era uma pessoa um bocado retraída. Independente, talvez, sei lá, enfim.

*E o doutor Deane não veio com a senhora nessa ocasião? Ele não estava aqui? A senhora estava sozinha?*

Não, não veio. Depois ele veio aqui fazer um estágio, voltou, e, quando nós fomos para o Ceará, nós fomos trabalhar no mesmo grupo... para a campanha do *gambiae*.

*A senhora lamentou ter que abandonar assim esse projeto de fazer o curso aqui?*

Não. Em parte, sim, mas em parte não, porque a gente ia se engajar num trabalho extremamente importante, muito interessante.

*Foi nesse momento que teve a briga.*

Foi. Nesse momento nós tivemos uma briga com o Evandro.

*Vocês acabaram ficando no outro projeto maior.*

O da Rockefeller. Fomos convidados pelo [Fred] Soper, que era o diretor da Rockefeller. Nós saímos... hoje nós lamentamos muito, mas tolices, bobagens... Nós fomos convidados pelo Soper – o Leônidas, eu, o irmão do Leônidas –^1^ para trabalhar no Serviço de Malária do Nordeste, e também na campanha contra o *gambiae*, que foi o serviço que realmente dominou, tomou conta da campanha. E aí também eu trabalhei num universo totalmente masculino, porque, fora secretárias, não tinha mulher, não. Eu nunca tive problemas. [Me sentia] à vontade completamente no ambiente de trabalho. Se bem que a gente sempre ouvia um zum-zum das pessoas. Por exemplo: criticavam que eu tinha um salário maior do que os vários homens. Também, mais uma vez, eu não dava a menor bola para essas coisas, realmente não dava. Engraçado: eu sempre soube, sempre tive ideia e sempre senti um pouquinho uma certa discriminação, mas eu nunca me senti diretamente discriminada [...]

*Era uma coisa vaga, difusa.*

Difusa, eu não sei se é porque eu não dava bola mesmo ou qualquer coisa... Agora, que há discriminação, há, há até hoje... nunca foi um obstáculo, nunca foi. Talvez um pouco... não posso dizer isso... talvez, um pouco de vaidade, orgulho, você sempre por cima, não dá bola porque você automaticamente põe... Numa posição inferior, eu não ligo... É lógico que há discriminação. A minha posição em relação a esses problemas é a seguinte: existe discriminação contra a mulher em todos os meios, existe mesmo, a gente sabe, não adianta querer negar; agora, a gente também não pode esperar que as coisas mudem de um momento para o outro. Esse sentimento de superioridade masculina é uma coisa tão arraigada na sociedade, tão antiga, que você não pode pensar que, de um momento para o outro, você vai mudar, não pode esperar isso aí. As coisas estão mudando. Agora, pessoalmente, eu sempre soube que havia, mas eu nunca me senti muito diretamente atingida, talvez porque eu nunca permiti que essa coisa me atingisse de uma maneira mais profunda... segui com os meus projetos.

### [O casamento, a maternidade e a carreira]

*[...] acredito que com a maternidade alguma coisa tenha mudado.*

É, mudou porque eu fui obrigada... quer dizer, durante um período eu não quis trabalhar fora.

*Foi uma opção [...] A gente falava do papel da maternidade, e a senhora provavelmente optou em ficar com a sua filha, não é?*

Quando meu marido foi convidado para trabalhar em São Paulo, na Faculdade de Medicina com o professor Samuel Pessoa – era um sonho dele –, ele aceitou. O professor Samuel Pessoa era uma figura que a gente respeitava muito e insistiu muito para me contratar também, mas eu não queria, porque eu queria justamente ter mais tempo para estar com a minha filha. Só depois que ela foi para a escola etc., eu comecei a trabalhar em tempo parcial...

*A senhora lamentou naquela ocasião não ter participado integralmente?*

Não, não lamentei, porque eu optei por isso. Várias colegas minhas daqui e de São Paulo pensavam diferente. Conheci várias que eram professoras, pesquisadoras etc., e algumas delas não tinham nenhum problema em deixar filhos pequenos com empregadas. Eu sempre fiz muita resistência a isso, sabe? Mas também, depois, eu comecei, aos poucos, a ter...

*Ter coragem de deixar com terceiros, não é? Talvez até pelo tipo de educação que a senhora mesma recebeu, em que o papel da mãe era tão presente[...]*

Não, eu não sei, sabe? Você sabe, eu não tenho opinião assim definitiva acerca de várias coisas, eu não tenho. Eu acho que, oh Deus! A vida da gente é curta, tudo é provisório neste mundo, a gente é provisório, tudo passa depressa demais. Eu acho que as pessoas têm uma certa liberdade de viver sua vida como bem lhes pareça, desde que não chateiem os outros. Eu não tenho, assim, opiniões definitivas a respeito das coisas. Eu não sei se é melhor a mulher dentro de casa, melhor a mulher fora de casa trabalhando, eu não sei. Eu sei que hoje é uma necessidade econômica porque, hoje, os homens que trabalham, na maior parte das vezes, não ganham o suficiente para sustentar a família. Essa é uma das coisas. E a outra coisa: por que não dar à mulher a oportunidade de realmente empregar os seus talentos, a sua inteligência em alguma coisa, mais do que só mexer panela, ora veja, por que não? Se a mulher quer ficar dentro de casa cuidando de filho, ótimo [...] A vivência leva a gente a certas posições, mas eu nunca fui intolerante. Eu, por exemplo, optei por ficar em casa cuidando da minha filha até ela ter uma certa capacidade de ficar na escola etc., porque foi uma opção. Eu não me senti obrigada a isso.

*Era mais uma valorização pessoal da maternidade?*

Sei lá se pensei assim dessa maneira, sei lá. Talvez fosse falta de confiança na empregada doméstica. Quando minha mãe morreu, minha filha tinha 2 anos. Quando minha mãe era viva, eu tinha confiança em deixar a minha filha com a minha mãe, aí eu ainda trabalhava fora. Depois que a minha mãe morreu, nessa ocasião, eu tinha até duas empregadas muito boas: uma babá e uma cozinheira. Apesar de depositar nelas uma certa confiança, não era absoluta. Eu não entregava a educação de uma criança a uma empregada [...]

*A senhora acha que a sua carreira ficou em parte prejudicada por esses anos de afastamento parcial?*

Logicamente que houve. Eu tenho no meu curriculum um espaço quase em branco. Durante esse tempo, eu aceitei fazer trabalhos de cursos. Naquele tempo, o Ministério da Saúde era Ministério da Educação e Saúde e organizava uns cursos de pós-graduação e me convidavam para dar cursos, para dar aulas, e eu dava. Eu fazia trabalhos também de tradução, de versão de trabalhos científicos para inglês, inglês para português e fazia curtas viagens para esses cursos etc. Mas eu não pude me dedicar às pesquisas, não pude publicar trabalhos. Nesse período, é um período relativamente grande, você não vê trabalhos publicados no meu curriculum, tem um espaço assim que não tem nada. Então, desse certo modo, sim, mas passou o tempo e isso ficou meio apagado.

*Porque até esse momento a sua carreira e a do doutor Deane eram muito misturadas.*

Muito misturadas.

*É nesse momento que há uma separação, talvez a primeira...*

É, do ponto de vista científico, é.

*Cada um faz um caminho independente.*

É, mas assim mesmo nós fizemos muita coisa juntos também: fomos para o Ceará fazer uns estudos, fomos convidados pelo Ministério da Saúde – fomos juntos nessa ocasião, temos uns trabalhos publicados juntos. Depois, mais recente também, mas isso já é outra... aí a minha filha era pequena, foi conosco... mas aí eu tinha uma irmã que deixou o emprego dela para ir conosco para tomar conta da menina.

*Isso foi uma facilidade, foi uma sorte, vocês poderem contar com esse apoio familiar. A irmã não tinha um vínculo assim muito forte com a profissão, pôde largar.*

Pois é. Então, foi isso. Mas depois vem um período negro na vida da gente... com a revolução de 1964.

[A ditadura civil-militar no Brasil e as implicações familiares]

*A gente não precisava dar esse pulo, mas, já que a senhora está mencionando, eu fiquei muito impressionada quando o doutor Deane contou que a senhora deixou de se aposentar, estava no fim da carreira, para ir ao encontro da filha. Que coisa tão forte, não é?*

Minha filha sofreu muito com essa coisa. Ela, como muitas jovens dessa época, se envolveu com política universitária. Envolveu, foi envolvida, e tal. Aquela coisa estúpida da repressão naquela época me obrigou a sair daqui do Brasil. Foi uma época muito dura, muito dura mesmo, para ela e para nós. Depois fomos para fora também, passamos vários anos fora, mas, enfim, tudo passa.

*É, mas esse realmente foi um corte muito violento no desenrolar assim das vidas.*

Não só nossa, mas de tantas pessoas. Nessa ocasião, eu tinha um pavor que a minha filha caísse na mão desses assassinos que andam à solta hoje por aí, não é? Várias pessoas que nós conhecemos, jovens, inteligentes, tão promissores, tão idealistas e tal, foram presos, torturados, alguns mortos. Não gosto de pensar nesse negócio, não, me revolta muito. Pra quê?

*O doutor Deane nos contou um episódio que imagino que tenha sido muito traumático na Argentina, quando ela esteve presa e vocês tinham aquela dificuldade toda...*

É, porque a polícia brasileira não era... Ela foi para Argentina numa tentativa de vir para o Brasil, mas, com pouca sorte, as coisas viraram também na Argentina, e a polícia brasileira tinha, logicamente, entrada franca nisso. Você sabe disso: a polícia brasileira não só mandava informações como... Negaram o passaporte, ela não podia entrar no Brasil sem ter passaporte; negaram o visto, ela tinha passaporte, mas negaram o visto. Depois ela foi presa. Nós fizemos um processo que foi até o Supremo Tribunal Militar e lá mantiveram a negativa de dar o visto [risos]. Porque ela era um elemento muito perigoso, depois, passada a história, a gente acha até graça. Então, um filho seu, que você está acostumado, pessoa generosa, bondosa e tudo, que não podia ver um animal maltratado... é muito perigosa [...]

Mas esteve presa em circunstâncias muito chatas, muito, muito chatas. Essas coisas passaram, como tudo. Um medo terrível, um medo danado. Na Argentina, nessa ocasião, realmente faziam desaparecer as pessoas.

*Eu estava falando com uma moça argentina [...] disse que praticamente não se encontra ninguém que não perdeu alguém nesses anos todos na Argentina. Disse que o povo era um povo muito triste, todo mundo muito marcado...*

Você viu esse filme, *A história oficial*? Interessante que nós íamos visitar a nossa filha na prisão e conhecemos lá uma companheira de prisão dela que tinha um filhinho, um bebezinho. A mãe dela e a sogra dessa moça iam visitá-la também, ao mesmo tempo. Eles faziam aquelas coisas humilhantes para a gente ficar em fila, revista, esse tipo de coisa. Então, a gente sempre conversava um pouco, e as duas iam visitar a filha de uma e nora da outra e o bebezinho. De vez em quando, davam licença para uma delas levar o bebezinho para casa. Um bebezinho loirinho, de olhos azuis – a moça era loura de olhos azuis. Levavam o bebezinho. Bom, depois nós conseguimos provar que não havia nada contra a nossa filha, e soltaram ela. Então, eles marcaram um ponto para a gente encontrar, à noite. Aí fomos encontrar, eles desviaram a nossa filha Luísa para uma outra prisão. Nos comunicaram que ia ficar presa com ordem direta da Presidência da República. Uma coisa completamente ilegal. Então, nós passamos a ir visitá-la lá, em condições péssimas, horríveis. Numa ocasião que nós estávamos lá na antessala para poder ver a filha, essas duas senhoras estavam lá, e, como nós tínhamos nos encontrado várias vezes na prisão, eu fiz menção de ir falar com elas e notei [...] que elas não queriam falar comigo [...] Bom, nós fomos visitar a menina, saímos, daqui a pouco dobramos uma esquina e escutamos aqueles passos assim atrás, elas vinham, as duas: “Olha, nós não quisemos falar com vocês porque não queríamos incriminar vocês mais do que o necessário, mas nós estamos aqui para saber notícias do nosso neto, porque eles sumiram com o garotinho”. Então, elas nos contaram o seguinte: a moça que estava presa, a mãe do menino [...] tinha tido a criança já na prisão, e o marido dela, que era filho da outra dona, não conhecia o garoto, mas elas sabiam onde ele estava – ele estava escondido. Elas sabiam onde ele estava e conseguiam se comunicar de vez em quando com ele. Passou o tempo, e o rapaz, doido para conhecer o filho, pedia sempre para levarem o garoto lá para ele conhecer e tal, e elas resistiam. Mas, um dia, elas acharam que a vigilância tinha diminuído, então se arriscaram a levar o garotinho para o pai conhecer. A polícia estava atrás, chegou na hora, prendeu o rapaz e levaram a criança, e elas estavam loucas para descobrir para onde eles tinham levado a criança. Até hoje eu não sei se eles chegaram a descobrir a criança. Quer dizer, foi a história de *A história oficial*. Um negócio terrível, não é? A capacidade que tem o indivíduo humano de ser cruel, é incrível, incrível.

## Data Availability

https://basearch.coc.fiocruz.br/index.php/depoimento-maria-jose-deane.

## NOTAS

1 O registro original foi feito em duas fitas cassete, totalizando 1h18min de gravação. A versão digitalizada do depoimento está disponível para os usuários interessados, e a mediação é realizada pela Sala de Consulta, do Departamento de Arquivo e Documentação, da Casa de Oswaldo Cruz. A transcrição integral está disponível em: https://basearch.coc.fiocruz.br/index.php/depoimento-maria-jose-deane. Acesso em: 9 set. 2024.

2 A Casa de Oswaldo Cruz vem desenvolvendo ações para ampliar a visibilidade das mulheres na ciência e nos arquivos científicos. Como exemplo, podemos citar o projeto “Mulheres na ciência e na saúde: digitalização e difusão dos arquivos pessoais de mulheres do acervo da Casa de Oswaldo Cruz/Fiocruz”, financiado pelo “Projeto Iberarchivos”, quando foram digitalizados dez arquivos de mulheres cientistas. Dos arquivos pessoais custodiados pela instituição, a maior parte tem homens como produtores.

3 Deve ser ressaltado aqui o momento de profundas mudanças na área da saúde pública após a criação, em 1930, do Ministério da Educação e Saúde, com Getúlio Vargas, cuja consolidação influenciou positivamente as instituições já existentes bem como as criadas naquela gestão. As ações de pesquisa para aprofundar estudos sobre as doenças brasileiras (doença de Chagas, malária, leishmaniose, entre outras) tiveram grande avanço naquele período.
